# Plasma GlycA, a Glycoprotein Marker of Chronic Inflammation, and All-Cause Mortality in Cirrhotic Patients and Liver Transplant Recipients

**DOI:** 10.3390/ijms26020459

**Published:** 2025-01-08

**Authors:** Yakun Li, Mateo Chvatal-Medina, Maria Camila Trillos-Almanza, Margery A. Connelly, Han Moshage, Stephan J. L. Bakker, Vincent E. de Meijer, Hans Blokzijl, Robin P. F. Dullaart

**Affiliations:** 1 Department of Gastroenterology and Hepatology, University Medical Center Groningen, University of Groningen, P.O. Box 30001, 9700 RB Groningen, The Netherlands; m.chvatal-medina@umcg.nl (M.C.-M.); m.c.trillos.almanza@umcg.nl (M.C.T.-A.); a.j.moshage@umcg.nl (H.M.); h.blokzijl@umcg.nl (H.B.); 2 Labcorp, 100 Perimeter Park, Morrisville, NC 27560, USA; connem5@labcorp.com; 3 Division of Nephrology, Department of Internal Medicine, University Medical Center Groningen, University of Groningen, P.O. Box 30001, 9700 RB Groningen, The Netherlands; s.j.l.bakker@umcg.nl; 4 Division of Hepato-Pancreato-Biliary Surgery and Liver Transplantation, Department of Surgery, University Medical Center Groningen, University of Groningen, P.O. Box 30001, 9700 RB Groningen, The Netherlands; v.e.de.meijer@umcg.nl; 5 Division of Endocrinology, Department of Internal Medicine, University Medical Center Groningen, University of Groningen, P.O. Box 30001, 9700 RB Groningen, The Netherlands

**Keywords:** chronic inflammation, liver cirrhosis, GlycA, hs-CRP, liver transplantation, mortality

## Abstract

Low-grade chronic inflammation may impact liver disease. We investigated the extent to which circulating GlycA, a glycoprotein biomarker of low-grade inflammation, and high-sensitivity C-reactive protein (hs-CRP) are altered in patients with cirrhosis and liver transplant recipients (LTRs) and examined their associations with all-cause mortality. Plasma GlycA (nuclear magnetic resonance spectroscopy) and hs-CRP (nephelometry) were assessed in 129 patients with cirrhosis on the waiting list for liver transplantation and 367 LTRs (TransplantLines cohort study; NCT03272841) and compared with 4837 participants from the population-based PREVEND cohort. GlycA levels were lower, while hs-CRP levels were higher in patients with cirrhosis compared to PREVEND participants (*p* < 0.001). Notably, GlycA increased, but hs-CRP decreased after transplantation. In LTRs, both GlycA and hs-CRP levels were higher than in PREVEND participants (*p* < 0.001). Survival was impaired in patients with cirrhosis and LTRs with the highest GlycA and the highest hs-CRP tertiles. In Cox regression analysis, GlycA remained associated with mortality in cirrhotic patients after adjusting for potential confounders and for hs-CRP (HR per 1-SD increment: 2.34 [95% CI 1.07–5.13]), while the association with hs-CRP after adjusting was lost. In LTRs, both GlycA and hs-CRP were also associated with mortality (adjusted HR: 1.60 [95% CI: 1.2–2.14] and 1.64 [95% CI: 1.08–2.51], respectively) but not independent of each other. GlycA increases while hs-CRP decreases after liver transplantation. Both inflammatory markers may be associated with all-cause mortality in cirrhotic patients and LTRs, while the association for GlycA seems at least as strong as that for hs-CRP.

## 1. Introduction

Cirrhosis represents the final phase of chronic liver disorders, where liver function deteriorates irreversibly, leading to severe complications and high mortality [1,2]. The primary treatment for severe cirrhosis is liver transplantation (LT), which offers a chance for a cure [3,4]. However, despite the success of LT in prolonging life expectancy, recipients continue to face major health risks, including increased mortality [4,5]. These persistent challenges are partly attributed to cirrhosis-associated immune dysfunction, which is increasingly recognized as a critical factor influencing clinical outcomes in patients with liver diseases [6,7,8]. This dysfunction involves two different immune phenotypes: low-grade systemic inflammation and high-grade systemic inflammation, with recent research increasingly focusing on the low-grade systemic inflammatory response [6].

To date, various biomarkers of enhanced low-grade inflammation have been utilized to measure inflammation and predict outcomes in liver disease. High-sensitivity C-reactive protein (hs-CRP) is a widely recognized systemic inflammation marker. Elevated levels of hs-CRP have been associated with worse outcomes in patients with liver disease [9,10,11,12,13,14], reflecting ongoing inflammation and cellular injury. Although hs-CRP is a useful indicator, it may not capture the full scope of low-grade inflammatory activities, especially in chronic conditions like cirrhosis or in assessing the health of transplant recipients. This highlights a crucial need for more comprehensive biomarkers that can better represent the extensive inflammatory activities in these patients.

Recently, GlycA has emerged as a novel marker of low-grade systemic inflammation [15]. GlycA is determined using nuclear magnetic resonance (NMR) spectroscopy. The signal comes from N-acetyl methyl groups mostly bound to acute-phase proteins, mainly α1-acid glycoprotein, haptoglobin, α1-antitrypsin, α1-antichymotrypsin, and transferrin [15]. Although GlycA and hs-CRP are found to be interrelated [16], GlycA seems to be a less variable biomarker of low-grade systemic inflammation [15]. This stability is likely due to its reflection of the acute-phase protein glycosylation process, providing a broader assessment of the inflammatory state.

Previously, GlycA has been proven relevant in diseases such as cardiovascular disorders and diabetes, where it correlates with long-term inflammation and predicts negative health outcomes [17,18]. However, so far, the potential role of GlycA in patients with cirrhosis and among liver transplant recipients (LTRs) remains unexplored.

We therefore initiated the present study on plasma concentrations of GlycA, along with hs-CRP, in patients with cirrhosis on the waiting list for LT and those who have received LT. We aimed to investigate: (1) to which extent plasma GlycA and hs-CRP are altered in cirrhosis and post-transplantation, (2) the determinants of plasma GlycA and hs-CRP in cirrhosis patients and LTRs, and (3) the associations of GlycA and hs-CRP with all-cause mortality in these patient groups.

## 2. Results

### 2.1. Comparison of Baseline Clinical Characteristics Between Patients with Cirrhosis, Liver Transplant Recipients, and PREVEND Participants

There were 129 patients with cirrhosis and 367 LTRs who participated in this study. Their clinical and laboratory characteristics were compared with 4837 participants from the Prevention of Renal and Vascular End-stage Disease (PREVEND) study (Table 1). The median age was higher in the cirrhotic and LTR groups compared to the PREVEND group (*p* < 0.001), with no significant difference between the cirrhotic and LTR groups (*p* = 0.65). Male predominance was noted in both the cirrhotic and LTR groups compared to the PREVEND cohort (*p* < 0.001), while differences between the cirrhotic patients and LTRs were not significant (*p* = 0.19). Cirrhotic patients had the highest body mass index (BMI) (*p* < 0.001). Lifestyle factors varied, with only 12.4% of cirrhotic patients and 9% of LTRs currently smoking compared to 27.3% in PREVEND (*p* < 0.001); alcohol consumption was also lower in cirrhotic patients. Diabetes prevalence was substantially higher in both cirrhotic and LTR groups vs. PREVEND (*p* < 0.001). Use of glucose-lowering and antihypertensive drugs was higher in cirrhotic and LTRs (*p* < 0.001). Total cholesterol, low-density lipoprotein (LDL) cholesterol, high-density lipoprotein (HDL) cholesterol, and triglyceride levels were all significantly different among the groups (*p* < 0.001), with cirrhotic patients typically showing lower levels than LTRs and PREVEND participants.

hs-CRP was elevated in cirrhotic patients and to a lesser extent in LTRs compared to PREVEND participants (Table 1; *p* < 0.001). Remarkably, GlycA was lower in cirrhotic patients than in the other groups (*p* < 0.001). Applying propensity score matching for age, sex, BMI, blood pressure, smoking status, history of cardiovascular disease (CVD), diabetes, and usage of glucose-lowering, lipid-lowering, and antihypertensive drugs, 111 and 338 participants from the PREVEND cohort were, respectively, matched for subsidiary analyses with the cirrhotic and LTR groups. This analysis confirmed that hs-CRP levels were elevated in both the cirrhotic patients and LTRs compared to their matched PREVEND participants (10 vs. 1.9 mg/L and 2 vs. 1.2 mg/L, respectively; *p* < 0.001 for both comparisons). Again, GlycA was lower in cirrhotic patients than in LTRs and PREVEND participants, whereas GlycA was elevated in LTRs compared to PREVEND participants (317 vs. 346 µmol/L and 371 vs. 347.5 µmol/L, respectively; *p* < 0.01 for both comparisons).

**Figure 1:**
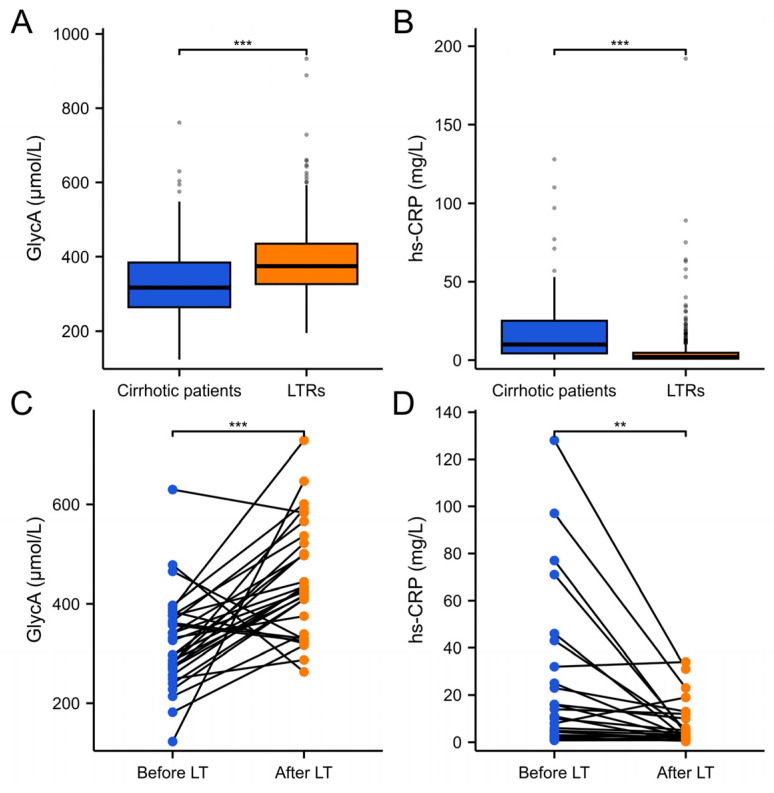
Changes in plasma GlycA and high-sensitivity C-reactive protein (hs-CRP) levels in cirrhotic patients and liver transplant recipients (LTRs). (**A**) Comparison of plasma GlycA levels between cirrhotic patients and LTRs (unpaired). (**B**) Comparison of hs-CRP levels between cirrhotic patients and LTRs (unpaired), (**C**) Paired analysis of GlycA levels in a subset of 30 patients studied twice, that is, before and after liver transplantation. (**D**) Paired analysis of hs-CRP levels in a subset of 23 patients studied twice, that is, before and after liver transplantation. GlycA levels significantly increase after LT, while hs-CRP levels show a significant decrease. *p* < 0.001 (***), *p* < 0.01 (**).

Figure 1A,B show plasma GlycA and hs-CRP levels in cirrhotic patients and in LTRs. GlycA measurements, both before and after the transplant, were available for 30 patients, and hs-CRP levels for 23 patients. For these individuals, the GlycA concentration increased (Figure 1C; *p* < 0.001), contrasting the decrease in hs-CRP (Figure 1D; *p* < 0.01).

Supplemental Table S1 shows baseline characteristics of cirrhotic patients and LTRs, categorized by tertiles of plasma GlycA levels. In cirrhotic patients, those in the highest GlycA tertile had significantly lower BMI and reduced HDL cholesterol, as well as elevated triglycerides and hs-CRP compared to those in lower tertiles (Supplemental Table S1A). For LTRs, higher GlycA tertiles were associated with older age; higher BMI; total cholesterol, LDL cholesterol, and triglycerides; and lower estimated glomerular filtration rate (eGFR). Additionally, significant differences were observed in the use of sirolimus, azathioprine, and liver function markers, such as total bilirubin, gamma-glutamyl transferase (GGT), and alkaline phosphatase (ALP), with increasing GlycA levels (Supplemental Table S1B).

### 2.2. Associations of GlycA and Hs-CRP with Clinical and Laboratory Variables in Patients with Cirrhosis and Liver Transplant Recipients

**Figure 2:**
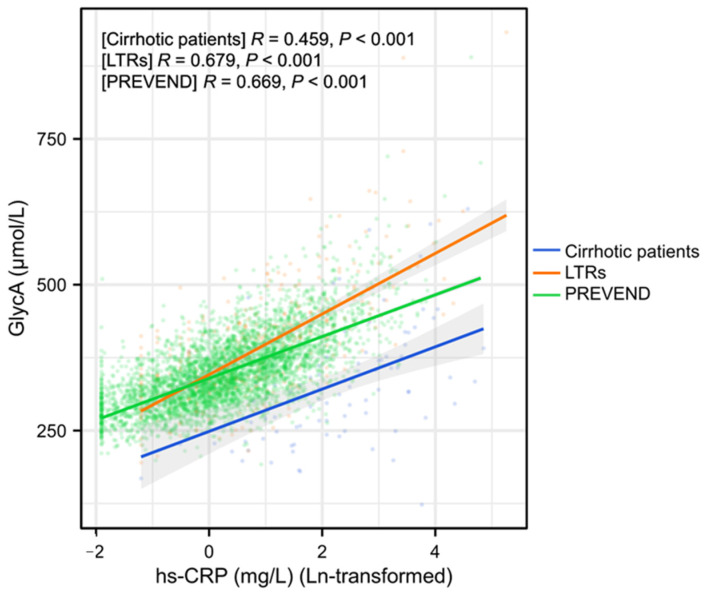
The scatter plot illustrates the positive correlation between plasma GlycA levels and high-sensitivity C-reactive protein (hs-CRP; ln-transformed) across three distinct populations: patients with cirrhosis, liver transplant recipients (LTRs), and PREVEND study participants. The colored lines represent the linear fit for each group, with shaded areas indicating the 95% confidence intervals. Cirrhotic patients (blue line) show a moderate correlation between GlycA and hs-CRP (R = 0.459, *p* < 0.001). LTRs (orange line) and PREVEND participants (green line) exhibit a stronger correlation (R = 0.679 and R = 0.669, respectively; both *p* < 0.001).

A strong positive correlation was observed between plasma GlycA and hs-CRP levels in LTRs (*R* = 0.679) and PREVEND participants (*R* = 0.669). In cirrhotic patients, this relationship was weaker (*R* = 0.459) (Figure 2). To disclose whether the relationship between GlycA and hs-CRP was different in cirrhotic patients than in LTRs and in PREVEND participants, we compared the regression lines between these groups. The results indicated that the interaction between hs-CRP and the group was statistically significant (*p* < 0.001), suggesting that the slopes of the regression lines differ significantly among the three groups. The main group effect was significant (*p* < 0.001), indicating differences in the intercepts.

Univariable and multivariable linear regression analyses in cirrhotic patients and LTRs identified significant associations between GlycA, hs-CRP, and various clinical and laboratory parameters (Supplemental Tables S2 and S3). In cirrhotic patients, GlycA was inversely associated with BMI and HDL cholesterol and positively with total cholesterol, LDL cholesterol, triglycerides, GGT, ALP, and hs-CRP in univariable analysis. Multivariable analysis confirmed positive associations with LDL cholesterol, triglycerides, and hs-CRP (Supplemental Table S2). For hs-CRP, univariable analysis showed an inverse association with HDL cholesterol and positive associations with Child–Turcotte–Pugh (CTP) stage, Model for End-stage Liver Disease (MELD) score, and GlycA, with multivariable analysis confirming positive associations with MELD score and GlycA (Supplemental Table S2). In LTRs, GlycA was related to age, BMI, lipid-lowering and glucose-lowering drugs, antihypertensives, metabolic dysfunction-associated steatotic liver disease (MASLD), use of mycophenolate, prednisone, sirolimus, tacrolimus (inverse), azathioprine (inverse), total cholesterol, LDL cholesterol, triglycerides, alanine aminotransferase (ALT), aspartate aminotransferase (AST), GGT, ALP, fasting glucose, glycated hemoglobin (HbA1c), and hs-CRP in univariable analysis. Multivariable analysis confirmed positive associations with age, MASLD, sirolimus use, LDL cholesterol, ALT, ALP, HbA1c, and hs-CRP (Supplemental Table S3). For hs-CRP, univariable analysis revealed positive associations with BMI, glucose-lowering drugs, prednisone use, sirolimus use, total cholesterol, ALT, AST, GGT, ALP, HbA1c, and GlycA, with GlycA remaining the strongest predictor in multivariable analysis (Supplemental Table S3).

### 2.3. Longitudinal Analysis of GlycA and Hs-CRP with All-Cause Mortality in Patients with Cirrhosis and Liver Transplant Recipients

Of the 129 cirrhotic patients, 29 (22.5%) died during follow-up of 140 (IQR 52–381) days. Of the 367 LTRs, 30 (8.2%) died during follow-up of 9 (IQR 3–17) years.

**Figure 3:**
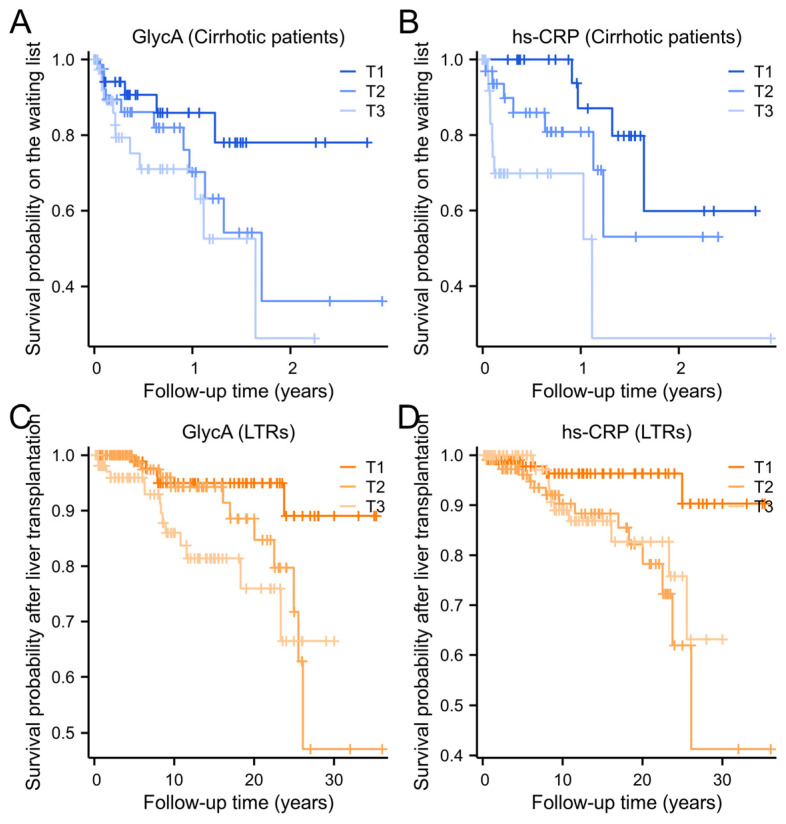
Kaplan–Meier survival curves for GlycA and hs-CRP levels in relation to all-cause mortality. (**A**) Survival probability for cirrhotic patients on the waiting list for liver transplantation according to GlycA tertiles (T1: <276 μmol/L; T2: 276–360 μmol/L; and T3: >360 μmol/L) (log-rank test T3 vs. T1, *p* = 0.033). (**B**) Survival probability on the waiting list for cirrhotic patients according to hs-CRP tertiles (T1: <5 mg/L; T2: 5–18 mg/L; and T3: >18 mg/L) (log-rank test T3 vs. T1, *p* = 0.003). (**C**) Post-liver transplant survival probability for liver transplant recipients (LTRs) according to GlycA tertiles (T1: <339 μmol/L; T2: 339–414 μmol/L; and T3: >414 μmol/L) (log-rank test T3 vs. T1, *p* = 0.005). (**D**) Post-liver transplant survival probability for LTRs according to hs-CRP tertiles (T1: <1.2 mg/L; T2: 1.2–3.5 mg/L; and T3: >3.5 mg/L) (log-rank test T3 vs. T1, *p* = 0.024). LTR: liver transplant recipient; hs-CRP: high-sensitivity C-reactive protein.

Figure 3 presents the Kaplan–Meier curves for all-cause mortality of cirrhotic patients and LTRs stratified by tertiles of GlycA and hs-CRP levels. In cirrhotic patients, higher GlycA (Figure 3A) and hs-CRP (Figure 3B) tertiles were associated with lower survival probabilities. Specifically, in GlycA tertiles, the survival probability was significantly lower in T3 compared to T1 (log-rank test, *p* = 0.033). Similarly, for hs-CRP, T3 showed a lower survival probability compared to T1 (log-rank test, *p* = 0.003).

In Cox proportional hazards regression analyses, both GlycA and hs-CRP were associated with increased all-cause mortality risk in cirrhotic patients (Table 2, Model 1, HR per 1-SD increment in GlycA: 1.73 [95% CI: 1.15–2.60], *p* = 0.009; HR per 1-SD increment in hs-CRP: 1.74 [95% CI: 1.23–2.47], *p* = 0.002). After adjusting for age and sex (Table 2, Model 2) and MELD scores (Table 2, Model 3), GlycA remained significantly associated with mortality, while the significance of hs-CRP diminished. With further adjustments for LDL cholesterol and triglycerides, the association with GlycA remained (Table 2, Model 4, HR per 1-SD increment: 2.76 [95% CI: 1.53–4.99], *p* = 0.001), while hs-CRP became non-significant. Even after adjusting for hs-CRP, GlycA remained significantly associated with mortality (Table 2, Model 5, HR per 1-SD increment: 2.34 [95% CI: 1.07–5.13], *p* = 0.033), whereas hs-CRP showed no significant association when adjusted for GlycA (*p* = 0.38).

In LTRs, higher GlycA (Figure 3C) and hs-CRP (Figure 3D) tertiles were associated with compromised post-transplantation survival. For GlycA, T3 showed significantly lower survival compared to T1 (log-rank test, *p* = 0.005). Similarly, for hs-CRP, both T2 and T3 demonstrated a higher risk compared to T1 (log-rank test, *p* = 0.003 and *p* = 0.024, respectively). Cox regression analyses in LTRs revealed significant associations of GlycA and hs-CRP with increased all-cause mortality in the crude model (Table 3, Model 1, HR per 1-SD increment in GlycA: 1.69 [95% CI: 1.29–2.21], *p* < 0.001; HR per 1-SD increment in hs-CRP: 1.25 [95% CI: 1.09–1.44], *p* = 0.002) and after adjusting for age, sex, and MASLD (Table 3, Models 2 and 3). However, when adjusted GlycA for hs-CRP or vice versa, neither GlycA nor hs-CRP remained significantly associated with mortality (Table 3, Model 4).

In a sensitivity analysis, excluding participants with a hs-CRP level >10 mg/L, point estimates of GlycA and hs-CRP with mortality in cirrhotic patients (*n* = 43; 6 deaths) were comparable to those in the whole group but did not reach significance (Supplemental Table S4). Also, in LTRs, point estimates of GlycA and hs-CRP with mortality were comparable to those found in the whole group (*n* = 305; 25 deaths). However, in LTRs, the association of GlycA with mortality was significant in the crude model (HR per 1-SD increment: 1.75 [95% CI: 1.06–2.90], *p* = 0.029). In LTRs, the association of hs-CRP with mortality was non-significant in all models.

## 3. Discussion

The present study has demonstrated that plasma GlycA concentrations were unexpectedly lower, whereas hs-CRP levels were considerably higher in patients with cirrhosis compared to the levels found in the general population. Following liver transplantation, GlycA levels increased by 38%, while hs-CRP levels decreased by 74%. In LTRs, both GlycA and hs-CRP levels were elevated compared to the control cohort. Furthermore, both GlycA and hs-CRP were associated with all-cause mortality in both cirrhotic patients and LTRs, with the association of GlycA with mortality being stronger than that of hs-CRP in cirrhotic patients. Collectively, these findings may suggest that the extent to which GlycA is associated with all-cause mortality in patients with cirrhosis and LTRs is at least as strong as that for hs-CRP.

We found an independent association of GlycA with LDL cholesterol and triglycerides, which are known to progressively decline with worsening of liver function [19], whereas hs-CRP was associated with the MELD score in multivariable analysis. Combined, these findings align with an effect of progressive liver failure on GlycA and hs-CRP. However, despite the positive relationship between GlycA and hs-CRP [16,20], as now also found in cirrhosis, we observed a notable discrepancy between these inflammation markers in cirrhotic patients, with hs-CRP levels being higher, while GlycA levels are lower compared to the general population. In fact, for a given hs-CRP, GlycA levels were lower in cirrhotic patients than in LTRs and PREVEND participants.

hs-CRP, an acute-phase protein mainly produced by the liver in response to inflammatory cytokines [21], is known to increase in patients with severe liver cirrhosis due to heightened inflammatory responses [22]. However, GlycA is derived from the glycosylation of acute-phase proteins, a process that is also primarily regulated by the liver [23]. Although both hs-CRP and GlycA are markers of acute-phase response, differences exist in their regulatory mechanisms and metabolic pathways. The synthesis of acute-phase glycoproteins, such as α1-acid glycoprotein and α1-antitrypsin, is more complex than that of hs-CRP, especially during the glycosylation step. Glycosylation requires a well-functioning liver with sufficient sugar supply, energy metabolism, and normal enzyme activity [24,25]. When liver function is severely compromised, the glycosylation process may be disrupted or less efficient, resulting in impaired acute-phase glycoprotein synthesis. Conceivably, ZIP8, a manganese transporter encoded by the *SLC39A8* gene that influences N-glycan branching, could be involved in lower circulating levels of GlycA in patients with cirrhosis. The SLC39A8 locus plays a crucial role in regulating the metabolism and transport of essential metal ions, particularly manganese and zinc, thereby affecting the N-glycosylation process [26]. ZIP8 is involved in the cellular uptake of these metal ions, both of which are critical for numerous biological processes, including immune function, antioxidant defense, and inflammation regulation [27,28]. Of further relevance, the *SLC39A8* locus may also confer protection against the progression of hepatic steatosis to steatohepatitis and fibrosis [27]. Evidently, further study is needed to delineate more precisely the mechanisms responsible for the lower GlycA levels in cirrhosis and the role of ZIP8/*SLC39A8* therein.

Following liver transplantation, we observed a significant increase in GlycA levels and a decrease in hs-CRP levels. As the new liver gradually regains normal function, it better regulates inflammatory responses, leading to reduced hs-CRP production. Even an intra-operative decrease in hs-CRP levels has also been shown to have the potential to predict overall post-transplant outcomes in LTRs [29]. However, hs-CRP may be less sensitive to chronic low-grade inflammation and could fail to detect ongoing systemic inflammation. Conversely, GlycA seems to be a more sensitive and less variable biomarker of low-grade systemic inflammation in non-cirrhotic individuals [15,30]. Interestingly, our study found elevated GlycA levels after LT, indicating the persistence of enhanced chronic low-grade inflammation. Recent research has highlighted the gut microbiota, specifically the composition and metabolites produced by gut bacteria, as a significant contributor to low-grade inflammation [31]. A key biomarker in this context is the gut-derived metabolite trimethylamine N-oxide (TMAO), which has been shown to be elevated in LTRs [32], potentially contributing to persistent low-grade inflammation. Another possible factor is the use of immunosuppressive agents. Our multivariable regression analysis found a positive association between GlycA levels and the use of sirolimus in LTRs. Immunosuppressive agents can impair gut barrier function, increasing intestinal permeability. When the gut barrier is compromised, bacteria, toxins, and inflammatory factors can more easily enter the bloodstream, exacerbating systemic inflammation [33,34]. Other factors, including age, diet, and pharmacological treatments, can significantly impact GlycA and hs-CRP levels and must be considered. These factors may interact with underlying inflammatory processes, contributing to the variability in these biomarkers. Future research that includes comprehensive data on these variables will be essential for fully understanding their roles in influencing GlycA and hs-CRP levels in LTRs.

In this study, we evaluated the association of hs-CRP and GlycA for all-cause mortality in cirrhotic patients and LTRs. While hs-CRP has been extensively studied and established as a biomarker for various diseases, GlycA is emerging as a novel marker, with most of the research focusing on its role in diabetes and CVD and its value to predict all-cause mortality in the general population and to confer reduced life expectancy [17,18,20,30,35]. To our knowledge, our study is the first to investigate GlycA specifically in a population of patients with cirrhosis and LTRs. Notably, GlycA could exhibit superior predictive capability for all-cause mortality in cirrhotic patients. After adjusting for hs-CRP, the association between GlycA and mortality remained significant, whereas the relationship between hs-CRP and mortality disappeared after adjusting for GlycA in the cirrhotic group, although in LTRs, the associations of GlycA and hs-CRP with mortality were similar. This suggests that GlycA might be a more robust predictor in the context of cirrhosis despite lower circulating levels. Moreover, it is important to highlight that GlycA is influenced by cardiovascular risk factors, suggesting that its levels may be modifiable through lifestyle interventions. In our study, we confirmed a relationship between GlycA and cardiometabolic risk factors, such as BMI, hyperglycemia, and total cholesterol, in univariable analysis, as found in non-liver disease populations [16,36]. Previous studies have demonstrated that lifestyle interventions can significantly lower GlycA levels. For instance, GlycA was markedly lowered in obese, prediabetic adolescent Latinos following lifestyle changes [37], and surgical weight loss was shown to lower GlycA levels as well [38]. Controlling modifiable risk factors can thus lead to a reduction in GlycA levels. This presents a promising opportunity for future therapeutic strategies aimed at lowering GlycA to improve patient outcomes. Further research is warranted to elucidate the underlying mechanisms linking GlycA with disease progression and to explore its potential as a predictive biomarker for clinical applications or perhaps even as a therapeutic target. Notably, however, the association of GlycA with mortality was independent of age and sex, suggesting that GlycA retains its prognostic value independent of age and gender, indicating its robustness as a biomarker.

Strengths of this study include combined GlycA and hs-CRP measurements in a considerable group of cirrhotic patients and LTRs with a detailed and standardized assessment of clinical and laboratory characteristics consequent to the TransplantLines Biobank and Cohort study set-up in comparison with data from the PREVEND study, which served as a large community-dwelling control cohort from the same region in the Netherlands. We included LTRs only who had received an LT at least one year before the collection of clinical and biochemical characterization and GlycA blood sampling to obviate confounding due to short-term LT-related complications. Moreover, a sensitivity analysis among cirrhotic patients and LTRs with an hs-CRP <10 mg/L, thereby essentially excluding participants with potential latent infections, defined by hs-CRP levels exceeding 10 mg/L, yielded similar hazard point estimates regarding associations of GlycA and hs-CRP with mortality, though not significant in most models due to lack of power. Our study’s limitations should also be acknowledged. As this was a single-center study, the external validity of the results may be restricted, reducing the ability to generalize the findings to broader or more diverse populations. Furthermore, the observational nature of our study and the retrospective setup of the TransplantLines Biobank and Cohort study may have induced insufficiently identified bias, precluding inference to causality. Additionally, our cohorts were predominantly composed of Western European ancestry, which may limit the generalizability of our findings to other ethnic groups.

## 4. Materials and Methods

### 4.1. Study Population

This study adhered to the STROBE (Strengthening the Reporting of Observational Studies in Epidemiology) guidelines [39].

Patients with cirrhosis and LTRs were recruited from the TransplantLines cohort, a prospective observational study based at the University Medical Center Groningen (UMCG), The Netherlands (NCT03272841) [40]. The TransplantLines study was approved by the UMCG Medical Ethics Committee (METc 2014/077). All participants provided written informed consent, and this study was conducted in compliance with the Declaration of Helsinki [41]. Exclusion criteria included inability to understand the Dutch language, cognitive impairment preventing comprehension of questionnaires or physical tests, prior retransplantation, and absence of GlycA measurements. LTRs who had received a liver transplant shorter than one year were excluded to obviate bias due to short-term complications and infections after transplantation.

For the control cohort, data were drawn from the PREVEND study [42,43], a population-dwelling cohort in the city of Groningen, The Netherlands. The PREVEND study, initially started between 1997 and 1998, has been described in detail elsewhere [42,43]. In brief, Groningen residents aged 28 to 75 years were invited to submit a morning urine sample and complete a demographic and cardiovascular history questionnaire. Pregnant women and those with insulin-dependent diabetes were excluded. Participants with a urinary albumin concentration of ≥10 mg/L, along with a randomly selected control group with lower concentrations, were invited for further evaluation. For this analysis, we focused on those who completed the second screening round between 2001 and 2003 and were confirmed to be free of liver disease (based on a questionnaire and medical records obtained from primary care physicians) and had available plasma GlycA measurements. The PREVEND study was approved by the UMCG Medical Ethics Committee (MEC96/01/022) and conducted according to the Declaration of Helsinki [41]. All participants from the TransplantLines and the PREVEND cohorts gave written informed consent. Supplemental Figure S1 shows a CONSORT flow chart of study participants from the TransplantLines and the PREVEND cohorts.

### 4.2. Data Collection and Clinical Measurements

The TransplantLines study is continuously collecting data from June 2015 onwards. For the current study, samples were collected up to June 2021. During outpatient visits, questionnaires and blood samples were obtained from all patients with standardized procedures [40]. At the time of blood collection, participants continued their regular medication. A standardized protocol was used to obtain anthropometric measurements. Patient information, including weight, height, BMI (calculated as weight divided by height squared), blood pressure, smoking status, alcohol consumption (standardized to 10 g per drink in the Netherlands), medication use (glucose and lipid-lowering drugs, antihypertensive medication, and immunosuppressants), and medical histories such as CVD and diabetes (defined as fasting plasma glucose >7.0 mmol/L, non-fasting plasma glucose >11.1 mmol/L, a self-reported diagnosis, or the use of glucose-lowering drugs), was extracted from the TransplantLines Biobank. Blood pressure was measured using an automatic device with multiple readings to ensure accuracy. The eGFR was calculated using the 2012 CKD Epidemiology Collaboration creatinine-based formula [44]. Additional review of electronic patient records of study participants was performed to obtain additional data concerning the etiology of liver disease. For cirrhotic patients, cirrhotic evaluations (based on imaging, histology, or transient elastography) and biochemical and clinical variables were used to compute the MELD and CTP scores to assess the severity of cirrhosis. The MELD score was calculated by serum total bilirubin, creatinine, and the international normalized ratio (INR) [45]. The CTP score was calculated by total bilirubin, serum albumin, INR, the presence of ascites, and hepatic encephalopathy [46]. For the TransplantLines cohort, data on mortality were obtained from electronic patient records and verified by the Dutch Central Bureau of Statistics.

In the PREVEND cohort, data were collected on demographics, lifestyle factors, anthropometric measurements, medical history, and medication use, which was combined with information from a pharmacy-dispensing registry as previously described [43].

### 4.3. Laboratory Measurements

Venous blood samples for both TransplantLines and PREVEND cohorts were collected after an overnight fast. Laboratory methods for PREVEND are reported as described in detail previously [43]. A panel of standardized laboratory assays, including serum ALT, AST, GGT, ALP, total bilirubin, albumin (only available in the TransplantLines cohort), serum creatinine, hemoglobin, thrombocytes, and leucocytes (only available in the TransplantLines cohort), HbA1c (only available in the TransplantLines cohort), and plasma glucose, were analyzed with standardized laboratory measurements and quality assessment control at the department of Laboratory Medicine of the University Medical Center Groningen, The Netherlands. hs-CRP was measured using nephelometry with a threshold of 0.18 mg/L (BNII; Dade Behring Diagnostics, Marburg, Germany).

Ethylenediaminetetraacetic acid (EDTA)-anticoagulated plasma samples were centrifuged at 1400× *g* for 15 min at 4 °C and then stored at −80 °C. Plasma samples were sent frozen at −80 °C to Labcorp (Morrisville, NC, USA) for analysis on the Vantera^®^ Clinical Analyzer. For lipoprotein profiles, plasma samples were prepared on board the instrument and automatically delivered to the flow probe in the NMR spectrometer’s magnetic field. Total cholesterol, HDL cholesterol, and triglycerides were measured employing the LP4 algorithm [19,47]. LDL cholesterol was calculated using the Friedewald equation.

GlycA levels were determined as previously described [15,17]. In short, the GlycA NMR signal comes from the N-acetyl methyl group protons of the N-acetylglucosamine moieties located on the bi-, tri-, and tetra-antennary branches of plasma glycoproteins, mainly α1-acid glycoprotein, haptoglobin, α1-antitrypsin, α1-antichymotrypsin, and transferrin. The coefficients of variation (CVs) for the GlycA assay range from 1.3% to 2.3%, and the GlycA signal remains stable in EDTA-plasma samples after prolonged storage at −80 °C.

### 4.4. Statistical Analysis

Statistical analyses were performed using IBM SPSS software (version 25.0, IBM Corp, Armonk, NY, USA) and R software (version 4.2.1, Vienna, Austria). A two-sided *p*-value of less than 0.05 was considered statistically significant. Continuous variables were presented as mean ± standard deviation (SD) for normally distributed data or as medians (interquartile range, IQR) for non-normally distributed data, while categorical variables were reported as frequencies and percentages.

The GlycA concentrations were divided into tertiles separately among patients with cirrhosis and LTRs. Baseline characteristics were compared between two groups (cirrhotic patients and LTRs) and across three groups (cirrhotic patients, LTRs, and PREVEND participants) or across tertiles. For normally distributed continuous variables, an independent *t*-test was used for two-group comparisons and one-way analysis of variance (ANOVA) for three-group comparisons. For non-normally distributed continuous variables, the Mann–Whitney U test was applied for two-group comparisons, while the Kruskal–Wallis test was used for three-group comparisons. Categorical variables between two groups were compared using the chi-square test or Fisher’s exact test, as appropriate, while three-group comparisons were assessed using the chi-square test. Paired data for GlycA and hs-CRP were analyzed using the Wilcoxon signed-rank test. For the propensity score matching analysis, covariates included age, gender, BMI, systolic and diastolic blood pressure (SBP and DBP), current smoking, history of CVD, diabetes, glucose-lowering drugs, lipid-lowering drugs, and antihypertensive medication. A match tolerance of less than or equal to 0.2 was applied to enhance the precision of the matching.

The relationship between GlycA and hs-CRP (Ln-transformed) levels among the cirrhotic group, LTRs, and PREVEND participants was assessed using Pearson correlation coefficients, with 95% confidence intervals (CIs). The generalized linear model was conducted to evaluate whether the relationship between GlycA and hs-CRP differs among cirrhotic patients, LTRs, and PREVEND participants. The analysis included both main effects and interaction terms to compare the slopes and intercepts of the regression lines for each group. Univariable and multivariable linear regression analyses were conducted to examine the associations between clinical or laboratory variables and GlycA or hs-CRP levels. Variables with significant associations in univariable analysis (*p* < 0.05) were included in the multivariable models. The variables identified as independently associated with GlycA were subsequently included in the Cox regression model to adjust for potential confounding effects.

To assess the survival distributions across tertiles of GlycA and hs-CRP levels, Kaplan–Meier curves were generated, and comparisons were made using the log-rank test. Survival time was defined from baseline until the date of the last examination that participants attended, the date of their death, or June 2021 (the final month of follow-up). Univariable and multivariable Cox proportional hazards regression analyses were conducted to explore the associations between GlycA, hs-CRP, and the risk of all-cause mortality. Multivariable Cox models were constructed using the enter method to include potential confounding factors simultaneously. Results were expressed as hazard ratios (HRs) with corresponding 95% confidence intervals (CIs). HRs of GlycA and hs-CRP-associated risk were reported per 1-SD increment. The proportional hazards assumption was tested to ensure that it was not violated. Sensitivity analyses were conducted to exclude participants with potential latent infections, defined by hs-CRP levels exceeding 10 mg/L.

## 5. Conclusions

In conclusion, GlycA increases, but hs-CRP decreases after live transplantation. We suggest that both inflammation markers may be associated with all-cause mortality in patients with cirrhosis and LTRs. The association of GlycA with mortality may be at least as strong as that with hs-CRP. These findings underscore the potential of GlycA, an NMR-based inflammation biomarker, for predicting all-cause mortality in cirrhotic patients and LTRs. GlycA may provide additional insights into risk assessment and long-term outcomes, emphasizing its role in identifying high-risk individuals and improving mortality prediction.

## Figures and Tables

**Table 1:** Clinical and laboratory characteristics, including GlycA and hs-CRP, in patients with cirrhosis, liver transplant recipients (LTRs), and PREVEND participants.

	Cirrhotic Patients (*n* = 129)	LTRs (*n* = 367)	PREVEND (*n* = 4837)	*p* Value *	*p* Value #
Age (years)	60 (52, 65)	59 (47, 67)	52 (44, 63)	<0.001	0.65
Sex				<0.001	0.19
Male, *n* (%)	84 (65.1%)	215 (58.6%)	2388 (49.4%)		
Female, *n* (%)	45 (34.9%)	152 (41.4%)	2449 (50.6%)		
BMI (kg/m^2^)	27.8 (24.8, 30.9)	25.9 (23, 29.5)	26.1 (23.7, 28.9)	<0.001	<0.001
SBP (mmHg)	115 (107, 130)	130 (120, 142)	123 (112, 137)	<0.001	<0.001
DBP (mmHg)	65 (59, 75)	79 (74, 87)	72 (67, 79)	<0.001	<0.001
Current smoking, *n* (%)	16 (12.4%)	33 (9%)	1321 (27.3%)	<0.001	0.26
Alcohol consumption (g/day)				<0.001	<0.001
0/rarely, *n* (%)	124 (96.1%)	267 (72.8%)	1697 (35.1%)		
0.1–10, *n* (%)	5 (3.9%)	67 (18.3%)	1218 (25.2%)		
10–30, *n* (%)	0 (0%)	25 (6.8%)	993 (20.5%)		
≥30, *n* (%)	0 (0%)	8 (2.2%)	929 (19.2%)		
Diabetes, *n* (%)	36 (27.9%)	104 (28.3%)	294 (6.1%)	<0.001	0.93
History of cardiovascular disease, *n* (%)	6 (4.7%)	28 (7.6%)	301 (6.2%)	0.418	0.25
Blood glucose-lowering drugs, *n* (%)	35 (27.1%)	77 (21%)	178 (3.7%)	<0.001	0.15
Lipid-lowering drugs, *n* (%)	19 (14.7%)	86 (23.4%)	458 (9.5%)	<0.001	0.037
Antihypertensives, *n* (%)	80 (62%)	167 (45.5%)	854 (17.7%)	<0.001	0.001
Etiology, *n* (%)			-	-	<0.001
MASLD	33 (25.6%)	34 (9.3%)			
Storage diseases	4 (3.1%)	33 (9%)			
Cholestatic liver diseases	33 (25.6%)	100 (27.2%)			
Alcohol	28 (21.7%)	48 (13.1%)			
Viral	12 (9.3%)	37 (10.1%)			
Autoimmune hepatitis	10 (7.8%)	19 (5.2%)			
Others	9 (7%)	96 (26.2%)			
Child–Turcotte–Pugh classification		-	-	-	-
A, *n* (%)	28 (21.7%)				
B, *n* (%)	63 (48.8%)				
C, *n* (%)	38 (29.5%)				
MELD score	15 (10,19)	-	-	-	-
Ascites, *n* (%)	48 (37.2%)				
Hepatic encephalopathy, *n* (%)	28 (21.7%)				
Esophageal variceal bleeding, *n* (%)	11 (8.5%)				
Total cholesterol (mmol/L)	3.2 (2.5, 4.1)	4.2 (3.6, 4.8)	5.3 (4.7, 6.1)	<0.001	<0.001
LDL cholesterol (mmol/L)	1.8 (1.3, 2.2)	2.1 (1.7, 2.5)	3.5 (2.9, 4.1)	<0.001	<0.001
HDL cholesterol (mmol/L)	0.9 (0.4, 1.2)	1.3 (1.1, 1.7)	1.2 (1.0, 1.4)	<0.001	<0.001
Triglycerides (mmol/L)	0.7 (0.5, 1.1)	1.3 (1.0, 1.8)	1.1 (0.8, 1.6)	<0.001	<0.001
Albumin (g/L)	31 (27, 36)	44 (42, 46)	-	-	<0.001
Fasting glucose (mmol/L)	6.2 (5, 8.2)	5.7 (5.2, 6.9)	4.8 (4.4, 5.3)	<0.001	0.35
HbA1c (%)	5.1 (4.5, 5.75)	5.4 (5.1, 6.1)	-	-	0.002
Serum creatinine (µmol/L)	73.2 (55.6, 95.7)	88.9 (74.6, 107.4)	83.2 (73.9, 92.4)	<0.001	<0.001
eGFR (mL/min/1.73 m^2^)	99.5 (75.5, 109.5)	77.9 (61.15, 97.6)	93.7 (81.6, 104.3)	<0.001	<0.001
Total bilirubin (µmol/L)	42 (23.3, 98.5)	10 (7, 14)	7 (5, 9)	<0.001	<0.001
ALT (U/L)	40 (28, 60)	25 (18, 36)	17 (13, 24)	<0.001	<0.001
AST (U/L)	54 (44, 84)	25 (20, 33)	22 (19, 26)	<0.001	<0.001
GGT (U/L)	95 (48.5, 150.5)	40.5 (21, 88.5)	24 (16, 38)	<0.001	<0.001
ALP (U/L)	141 (98.5, 213.5)	87 (69, 126.5)	66 (55, 79)	<0.001	<0.001
Hemoglobin (mmol/L)	6.8 ± 1.3	8.5 ± 1.2	8.5 ± 0.8	<0.001	<0.001
Thrombocytes (×10^9^/L)	112 (78, 148.5)	197 (153.8, 246.5)	-	-	<0.001
Leucocytes (×10^9^/L)	4.9 (3.6, 7.6)	6.1 (4.8, 7.6)	-	-	<0.001
hs-CRP (mg/L)	10 (4.3, 25)	2 (0.9, 4.7)	1.3 (0.6, 3.0)	<0.001	<0.001
GlycA (μmol/L)	317 (264, 385)	375 (326.5, 435)	344 (308, 388)	<0.001	<0.001

Data are presented as mean ± SD, median (IQR), or as proportions (*n*) with corresponding percentages (%). * *p* for comparison among three groups; # *p* for comparison between cirrhotic and LTR groups. LTRs: liver transplant recipients; PREVEND: Prevention of Renal and Vascular End-stage Disease; BMI: body mass index; SBP: systolic blood pressure; DBP: diastolic blood pressure; MASLD: metabolic dysfunction-associated steatotic liver disease; MELD: model for end-stage liver disease; LDL: low-density lipoprotein cholesterol; HDL: high-density lipoprotein cholesterol; HbA1c: hemoglobin A1c; eGFR: estimated glomerular filtration rate; ALT: alanine aminotransferase; AST: aspartate aminotransferase; GGT: gamma-glutamyl transferase; ALP: alkaline phosphatase; hs-CRP: high-sensitivity C-reactive protein.

**Table 2:** Cox regression analyses for associations between plasma GlycA and hs-CRP levels and the risk of all-cause mortality in cirrhotic patients.

	GlycA, Per 1 SD Increment		hs-CRP, Per 1 SD Increment	
	HR [95% CI]	*p* Value	HR [95% CI]	*p* Value
Model 1	1.73 [1.15–2.60]	0.009	1.74 [1.23–2.47]	0.002
Model 2	1.81 [1.18–2.78]	0.006	1.63 [1.14–2.33]	0.007
Model 3	1.70 [1.08–2.68]	0.021	1.46 [0.99–2.17]	0.056
Model 4	2.76 [1.53–4.99]	0.001	1.46 [0.99–2.16]	0.059
Model 5	2.34 [1.07–5.13]	0.033	1.22 [0.78–1.91]	0.38

Model 1, crude model. Model 2, model 1 with adjustment for age and sex. Model 3, model 2 with adjustment for MELD scores. Model 4, model 3 with adjustment for LDL cholesterol and triglycerides. Model 5, model 4 with adjustment for hs-CRP (for GlycA) or GlycA (for hs-CRP). hs-CRP: high-sensitivity C-reactive protein; MELD: model for end-stage liver disease; LDL: low-density lipoprotein.

**Table 3:** Cox regression analyses for associations between plasma GlycA and hs-CRP levels and the risk of all-cause mortality in liver transplant recipients.

	GlycA, Per 1 SD Increment		hs-CRP, Per 1 SD Increment	
	HR [95% CI]	*p* Value	HR [95% CI]	*p* Value
Model 1	1.69 [1.29–2.21]	<0.001	1.25 [1.09–1.44]	0.002
Model 2	1.59 [1.19–2.13]	0.002	1.24 [1.07–1.44]	0.004
Model 3	1.60 [1.19–2.14]	0.002	1.64 [1.08–2.51]	0.021
Model 4	1.45 [0.90–2.32]	0.124	1.06 [0.82–1.38]	0.652

Model 1, crude model. Model 2, model 1 with adjustment for age and sex. Model 3, model 2 with MASLD. Model 4, model 3 with adjustment hs-CRP (for GlycA) or GlycA (for hs-CRP). LTR, liver transplant recipient; hs-CRP: high-sensitivity C-reactive protein; MASLD: metabolic dysfunction-associated steatotic liver disease.

## Data Availability

Data are available upon reasonable request.

## Supplementary Materials

The following supporting information can be downloaded at: https://www.mdpi.com/article/10.3390/ijms26020459/s1.

## References

[1] Kaplan A., Fortune B., Ufere N., Brown R.S., Rosenblatt R. (2021). National Trends in Location of Death in Patients with End-Stage Liver Disease. Liver Transpl..

[2] Wahid N.A., Lee J., Kaplan A., Fortune B.E., Safford M.M., Brown R.S., Rosenblatt R. (2021). Medicaid Expansion Association with End-Stage Liver Disease Mortality Depends on Leniency of Medicaid Hepatitis C Virus Coverage. Liver Transpl..

[3] Miro J., Laguno M., Moreno A., Rimola A., Hospital Clinic Olt In Hiv Working Group (2006). Management of end stage liver disease (ESLD): What is the current role of orthotopic liver transplantation (OLT)?. J. Hepatol..

[4] Burra P., Becchetti C., Germani G. (2020). NAFLD and liver transplantation: Disease burden, current management and future challenges. JHEP Rep..

[5] Serrano M.T., Sabroso S., Esteban L.M., Berenguer M., Fondevila C., Lorente S., Cortés L., Sanchez-Antolin G., Nuño J., De La Rosa G. (2022). Mortality and Causes of Death After Liver Transplantation: Analysis of Sex Differences in a Large Nationwide Cohort. Transpl. Int..

[6] Albillos A., Martin-Mateos R., Van Der Merwe S., Wiest R., Jalan R., Álvarez-Mon M. (2022). Cirrhosis-associated immune dysfunction. Nat. Rev. Gastroenterol. Hepatol..

[7] Trebicka J., Amoros A., Pitarch C., Titos E., Alcaraz-Quiles J., Schierwagen R., Deulofeu C., Fernandez-Gomez J., Piano S., Caraceni P. (2019). Addressing Profiles of Systemic Inflammation Across the Different Clinical Phenotypes of Acutely Decompensated Cirrhosis. Front. Immunol..

[8] Tilg H., Moschen A.R. (2008). Insulin resistance, inflammation, and non-alcoholic fatty liver disease. Trends Endocrinol. Metab..

[9] Liu Y.-B., Ying J., Kuang S.-J., Jin H.-S., Yin Z., Chang L., Yang H., Ou Y.-L., Zheng J.-H., Zhang W.-D. (2015). Elevated Preoperative Serum Hs-CRP Level as a Prognostic Factor in Patients Who Underwent Resection for Hepatocellular Carcinoma. Medicine.

[10] Jamialahmadi T., Bo S., Abbasifard M., Sathyapalan T., Jangjoo A., Moallem S.A., Almahmeed W., Ashari S., Johnston T.P., Sahebkar A. (2023). Association of C-reactive protein with histological, elastographic, and sonographic indices of non-alcoholic fatty liver disease in individuals with severe obesity. J. Health Popul. Nutr..

[11] Zanetto A., Pelizzaro F., Campello E., Bulato C., Balcar L., Gu W., Gavasso S., Saggiorato G., Zeuzem S., Russo F.P. (2023). Severity of systemic inflammation is the main predictor of ACLF and bleeding in individuals with acutely decompensated cirrhosis. J. Hepatol..

[12] Pompili E., Baldassarre M., Bedogni G., Zaccherini G., Iannone G., De Venuto C., Pratelli D., Palmese F., Domenicali M., Caraceni P. (2024). Predictors of clinical trajectories of patients with acutely decompensated cirrhosis. An external validation of the PREDICT study. Liver Int..

[13] Turco L., Garcia-Tsao G., Magnani I., Bianchini M., Costetti M., Caporali C., Colopi S., Simonini E., De Maria N., Banchelli F. (2018). Cardiopulmonary hemodynamics and C-reactive protein as prognostic indicators in compensated and decompensated cirrhosis. J. Hepatol..

[14] Trebicka J., Fernandez J., Papp M., Caraceni P., Laleman W., Gambino C., Giovo I., Uschner F.E., Jimenez C., Mookerjee R. (2020). The PREDICT study uncovers three clinical courses of acutely decompensated cirrhosis that have distinct pathophysiology. J. Hepatol..

[15] Otvos J.D., Shalaurova I., Wolak-Dinsmore J., Connelly M.A., Mackey R.H., Stein J.H., Tracy R.P. (2015). GlycA: A Composite Nuclear Magnetic Resonance Biomarker of Systemic Inflammation. Clin. Chem..

[16] Connelly M.A., Otvos J.D., Shalaurova I., Playford M.P., Mehta N.N. (2017). GlycA, a novel biomarker of systemic inflammation and cardiovascular disease risk. J. Transl. Med..

[17] Bourgonje A.R., Van Der Vaart A., Gruppen E.G., Van Goor H., Bakker S.J.L., Connelly M.A., Van Dijk P.R., Dullaart R.P.F. (2023). Plasma levels of GlycA, a pro-inflammatory glycoprotein biomarker, associate with an increased risk of microvascular complications in patients with type 2 diabetes (Zodiac-62). Endocrine.

[18] Akinkuolie A.O., Buring J.E., Ridker P.M., Mora S. (2014). A Novel Protein Glycan Biomarker and Future Cardiovascular Disease Events. J. Am. Heart Assoc..

[19] Van Den Berg E.H., Flores-Guerrero J.L., Gruppen E.G., Garcia E., Connelly M.A., De Meijer V.E., Bakker S.J.L., Blokzijl H., Dullaart R.P.F. (2022). Profoundly Disturbed Lipoproteins in Cirrhotic Patients: Role of Lipoprotein-Z, a Hepatotoxic LDL-like Lipoprotein. J. Clin. Med..

[20] Gruppen E.G., Riphagen I.J., Connelly M.A., Otvos J.D., Bakker S.J.L., Dullaart R.P.F. (2015). GlycA, a Pro-Inflammatory Glycoprotein Biomarker, and Incident Cardiovascular Disease: Relationship with C-Reactive Protein and Renal Function. PLoS ONE.

[21] Zhou H.-H., Tang Y.-L., Xu T.-H., Cheng B. (2024). C-reactive protein: Structure, function, regulation, and role in clinical diseases. Front. Immunol..

[22] Lai C.-Y., Cheng S.-B., Lee T.-Y., Liu H.-T., Huang S.-C., Huang Y.-C. (2018). Possible Synergistic Effects of Glutathione and C-Reactive Protein in the Progression of Liver Cirrhosis. Nutrients.

[23] Callewaert N., Vlierberghe H.V., Hecke A.V., Laroy W., Delanghe J., Contreras R. (2004). Noninvasive diagnosis of liver cirrhosis using DNA sequencer–based total serum protein glycomics. Nat. Med..

[24] Scheper A.F., Schofield J., Bohara R., Ritter T., Pandit A. (2023). Understanding glycosylation: Regulation through the metabolic flux of precursor pathways. Biotechnol. Adv..

[25] McCarthy C., Saldova R., Wormald M.R., Rudd P.M., McElvaney N.G., Reeves E.P. (2014). The Role and Importance of Glycosylation of Acute Phase Proteins with Focus on Alpha-1 Antitrypsin in Acute and Chronic Inflammatory Conditions. J. Proteome Res..

[26] Noel M., Chasman D.I., Mora S., Otvos J.D., Palmer C.D., Parsons P.J., Smoller J.W., Cummings R.D., Mealer R.G. (2023). The Inflammation Biomarker GlycA Reflects Plasma N-Glycan Branching. Clin. Chem..

[27] Nebert D.W., Liu Z. (2019). SLC39A8 gene encoding a metal ion transporter: Discovery and bench to bedside. Hum. Genom..

[28] Lin W., Vann D.R., Doulias P.-T., Wang T., Landesberg G., Li X., Ricciotti E., Scalia R., He M., Hand N.J. (2017). Hepatic metal ion transporter ZIP8 regulates manganese homeostasis and manganese-dependent enzyme activity. J. Clin. Investig..

[29] Chung H.S., Kim E.S., Park J.H., Park C.S. (2015). Prediction of Gross Post-Transplant Outcomes Based on the Intra-Operative Decline in C-Reactive Protein in Living Donor Liver Transplantation. Transplant. Proc..

[30] Gruppen E.G., Kunutsor S.K., Kieneker L.M., Van Der Vegt B., Connelly M.A., De Bock G.H., Gansevoort R.T., Bakker S.J.L., Dullaart R.P.F. (2019). GlycA, a novel pro-inflammatory glycoprotein biomarker is associated with mortality: Results from the PREVEND study and meta-analysis. J. Intern. Med..

[31] Tilg H., Zmora N., Adolph T.E., Elinav E. (2020). The intestinal microbiota fuelling metabolic inflammation. Nat. Rev. Immunol..

[32] Trillos-Almanza M.C., Chvatal-Medina M., Connelly M.A., Moshage H., TransplantLines Investigators Bakker S.J.L., De Meijer V.E., Blokzijl H., Dullaart R.P.F. (2024). Circulating Trimethylamine-N-Oxide Is Elevated in Liver Transplant Recipients. Int. J. Mol. Sci..

[33] Han Y., Wu L., Ling Q., Wu P., Zhang C., Jia L., Weng H., Wang B. (2021). Intestinal Dysbiosis Correlates with Sirolimus-induced Metabolic Disorders in Mice. Transplantation.

[34] Faucher Q., Jardou M., Brossier C., Picard N., Marquet P., Lawson R. (2022). Is Intestinal Dysbiosis-Associated with Immunosuppressive Therapy a Key Factor in the Pathophysiology of Post-Transplant Diabetes Mellitus?. Front. Endocrinol..

[35] Duprez D.A., Otvos J., Sanchez O.A., Mackey R.H., Tracy R., Jacobs D.R. (2016). Comparison of the Predictive Value of GlycA and Other Biomarkers of Inflammation for Total Death, Incident Cardiovascular Events, Noncardiovascular and Noncancer Inflammatory-Related Events, and Total Cancer Events. Clin. Chem..

[36] Fizelova M., Jauhiainen R., Kangas A.J., Soininen P., Ala-Korpela M., Kuusisto J., Laakso M., Stančáková A. (2017). Differential Associations of Inflammatory Markers with Insulin Sensitivity and Secretion: The Prospective METSIM Study. J. Clin. Endocrinol. Metab..

[37] Olson M.L., Rentería-Mexía A., Connelly M.A., Vega-López S., Soltero E.G., Konopken Y.P., Williams A.N., Castro F.G., Keller C.S., Yang H.P. (2019). Decreased GlycA after lifestyle intervention among obese, prediabetic adolescent Latinos. J. Clin. Lipidol..

[38] Manmadhan A., Lin B., Zhong J., Parikh M., Berger J.S., Fisher E.A., Heffron S.P. (2019). Elevated GlycA in severe obesity is normalized by bariatric surgery. Diabetes Obes. Metab..

[39] Von Elm E., Altman D.G., Egger M., Pocock S.J., Gøtzsche P.C., Vandenbroucke J.P. (2014). The Strengthening the Reporting of Observational Studies in Epidemiology (STROBE) Statement: Guidelines for reporting observational studies. Int. J. Surg..

[40] Eisenga M.F., Gomes-Neto A.W., Van Londen M., Ziengs A.L., Douwes R.M., Stam S.P., Osté M.C.J., Knobbe T.J., Hessels N.R., Buunk A.M. (2018). Rationale and design of TransplantLines: A prospective cohort study and biobank of solid organ transplant recipients. BMJ Open.

[41] World Medical Association (2013). World Medical Association Declaration of Helsinki: Ethical Principles for Medical Research Involving Human Subjects. JAMA.

[42] Borggreve S.E., Hillege H.L., Wolffenbuttel B.H.R., De Jong P.E., Bakker S.J.L., Van Der Steege G., Van Tol A., Dullaart R.P.F. (2005). The Effect of Cholesteryl Ester Transfer Protein −629C→A Promoter Polymorphism on High-Density Lipoprotein Cholesterol Is Dependent on Serum Triglycerides. J. Clin. Endocrinol. Metab..

[43] Kappelle P.J.W.H., Gansevoort R.T., Hillege J.L., Wolffenbuttel B.H.R., Dullaart R.P.F., on behalf of the PREVEND study group (2011). Apolipoprotein B/A-I and total cholesterol/high-density lipoprotein cholesterol ratios both predict cardiovascular events in the general population independently of nonlipid risk factors, albuminuria and C-reactive protein. J. Intern. Med..

[44] Inker L.A., Schmid C.H., Tighiouart H., Eckfeldt J.H., Feldman H.I., Greene T., Kusek J.W., Manzi J., Van Lente F., Zhang Y.L. (2012). Estimating Glomerular Filtration Rate from Serum Creatinine and Cystatin C. N. Engl. J. Med..

[45] Wiesner R., Edwards E., Freeman R., Harper A., Kim R., Kamath P., Kremers W., Lake J., Howard T., Merion R.M. (2003). Model for end-stage liver disease (MELD) and allocation of donor livers. Gastroenterology.

[46] Pugh R.N.H., Murray-Lyon I.M., Dawson J.L., Pietroni M.C., Williams R. (2005). Transection of the oesophagus for bleeding oesophageal varices. Br. J. Surg..

[47] Bedi S., Garcia E., Jeyarajah E., Shalaurova I., Perez-Matos M., Jiang Z., Dullaart R., Matyus S., Kirk W., Otvos J. (2020). Characterization of LP-Z Lipoprotein Particles and Quantification in Subjects with Liver Disease Using a Newly Developed NMR-Based Assay. J. Clin. Med..

