# Supplementary material for: Plasma GlycA, a Glycoprotein Marker of Chronic Inflammation, and All-Cause Mortality in Cirrhotic Patients and Liver Transplant Recipients
Source: Int J Mol Sci. 2025 Jan 8;26(2):459. doi: 10.3390/ijms26020459 (PMC11765328; doi:10.3390/ijms26020459)
Supplement: Supplementary file 1 [file ijms-26-00459-s001.zip › ijms-3375079-supplementary.pdf]

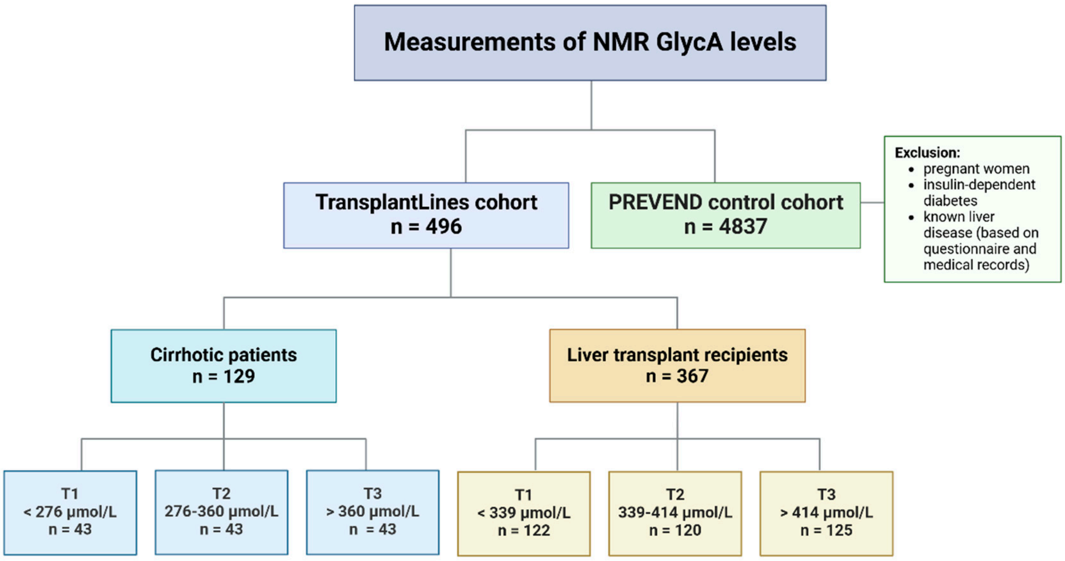

Supplemental Figure S1 CONSORT flow chart of the study population. NMR: Nuclear magnetic resonance spectroscopy. T1-T3: Tertiles of GlycA levels, 1 through 3.

Supplemental Table S1A Baseline characteristics of patients with cirrhosis, according to tertiles of plasma GlycA levels

	T1<br><276 µmol/L<br>(n=43)	T2<br>276-360 µmol/L<br>(n=43)	T3<br>>360 µmol/L<br>(n=43)	*P* value
Age (years)	60 (55, 65)	59 (53, 64)	60 (51, 65)	0.91
Sex				0.40
Male, n (%)	31 (72.1%)	28 (65.1%)	25 (58.1%)	
Female, n (%)	12 (27.9%)	15 (34.9%)	18 (41.9%)	
BMI (kg/m^2)	29.6 ± 4.9	28.7 ± 4.6	26.5 ± 4.4	0.008
SBP (mmHg)	116 (109, 135)	119 (107, 133)	111 (103, 124)	0.15
DBP (mmHg)	66 ± 10	67 ± 11	67 ± 13	0.83
Current smoking, n (%)	7 (16.3%)	5 (11.6%)	4 (9.3%)	0.61
Alcohol consumption (g/day)				0.44
0/rarely, n (%)	40 (93%)	42 (97.7%)	42 (97.7%)	

	T1<br><276 μmol/L<br>(n=43)	T2<br>276-360 μmol/L<br>(n=43)	T3<br>>360 μmol/L<br>(n=43)	*P* value
0.1-10, n (%)	3 (7%)	1 (2.3%)	1 (2.3%)	
Diabetes, n (%)	13 (30.2%)	15 (34.9%)	8 (18.6%)	0.22
History of cardiovascular disease, n (%)	1 (2.3%)	2 (4.7%)	3 (7%)	0.59
Blood glucose-lowering drugs, n (%)	12 (27.9%)	14 (32.6%)	9 (20.9%)	0.48
Lipid-lowering drugs, n (%)	5 (11.6%)	5 (11.6%)	9 (20.9%)	0.37
Antihypertensives, n (%)	23 (53.5%)	31 (72.1%)	26 (60.5%)	0.20
Etiology, n (%)				0.15
MASLD	10 (23.3%)	12 (27.9%)	11 (25.6%)	
storage diseases	3 (7%)	0 (0%)	1 (2.3%)	
Cholestatic liver diseases	6 (14%)	10 (23.3%)	17 (39.5%)	
Alcohol	13 (30.2%)	11 (25.6%)	4 (9.3%)	
Viral	6 (14%)	2 (4.7%)	4 (9.3%)	
Autoimmune hepatitis	3 (7%)	4 (9.3%)	3 (7%)	
Others	2 (4.7%)	4 (9.3%)	3 (7%)	
Child-Turcotte-Pugh classification				0.076
A, n (%)	4 (9.3%)	12 (27.9%)	12 (27.9%)	
B, n (%)	28 (65.1%)	18 (41.9%)	17 (39.5%)	
C, n (%)	11 (25.6%)	13 (30.2%)	14 (32.6%)	
MELD score	15 (12, 18)	13 (9.5, 18)	16 (11, 20)	0.25
Total cholesterol (mmol/L)	3.2 (2.5, 4.0)	3.1 (2.7, 3.9)	3.4 (2.4, 4.6)	0.72
LDL cholesterol (mmol/L)	1.6 (1.1, 2.1)	1.9 (1.4, 2.3)	1.9 (1.4, 2.7)	0.036
HDL cholesterol (mmol/L)	1.1 (0.7, 1.3)	1.0 (0.7, 1.2)	0.6 (0.1, 0.9)	< 0.001
Triglycerides (mmol/L)	0.5 (0.4, 0.8)	0.6 (0.5, 0.9)	1.1 (0.7, 1.4)	< 0.001
Albumin (g/L)	30 (27.5, 35)	32 (27, 36)	30.5 (26.8, 37.3)	0.87
HbA1c (%)	4.9 (4.6, 5.5)	5.4 (4.9, 6.0)	4.8 (4.1, 6.1)	0.34
Serum creatinine (μmol/L)	72.8 (55.6, 93.0)	71.7 (53.1, 99.8)	74.9 (61.9, 95.5)	0.78
eGFR (mL/min/1.73 m^2)	102.5 (81.5, 109.8)	99.7 (76.8, 108.1)	97.8 (73.2, 108.5)	0.52
Total bilirubin (μmol/L)	33 (24, 56.5)	39 (21.5, 64.25)	93 (25, 223.5)	0.086
ALT (U/L)	33 (25, 55.3)	32.5 (25.8, 45)	51 (38, 80)	0.004
AST (U/L)	51 (44.3, 63.5)	49.5 (37, 60)	81 (52, 118)	0.024
GGT (U/L)	50.5 (30.3, 95.8)	123 (73.5, 167.5)	113 (68, 284)	< 0.001
ALP (U/L)	126 (101, 153)	127 (86, 193.3)	210 (129, 305)	0.005
Fasting glucose (mmol/L)	6.5 (5.3, 8.5)	5.7 (4.825, 7.325)	6.25 (4.85, 8.7)	0.22
Hemoglobin (mmol/L)	6.7 ± 1.5	6.8 ± 1.1	7.1 ± 1.3	0.48
Thrombocytes (*10^9/L)	80 (64, 108.5)	117 (78, 138)	138 (113, 211)	< 0.001
Leucocytes (*10^9/L)	4.1 (3.6, 6.2)	4.2 (3.3, 5.9)	6.4 (4.5, 9.6)	0.007
hs-CRP (mg/L)	4.5 (2.4, 9)	11 (5.8, 21.3)	20.5 (10.3, 37)	< 0.001

Data are presented as mean ± SD, median (IQR) or as proportions (n) with corresponding percentages (%). BMI: body mass index; SBP: systolic blood pressure; DBP: diastolic blood pressure; MASLD: metabolic dysfunction-associated steatotic liver disease; MELD: model for end-stage liver disease; LDL: low-density lipoprotein cholesterol; HDL: high-density lipoprotein cholesterol; HbA1c: hemoglobin A1c; eGFR: estimated glomerular filtration rate; ALT: alanine aminotransferase; AST: aspartate aminotransferase; GGT: gamma-glutamyl transferase; ALP: alkaline phosphatase; hs-CRP: high-sensitivity C-reactive protein.

Supplemental Table S1B Baseline characteristics of liver transplant recipients, according to tertiles of plasma GlycA levels

	T1<br><339 μmol/L<br>(N=122)	T2<br>339-414 μmol/L<br>(N=120)	T3<br>>414 μmol/L<br>(N=125)	P value
Age (years)	51 (38, 62)	60 (50, 68)	63 (55, 67)	< 0.001
Sex				0.26
Male, n (%)	78 (63.9%)	70 (58.3%)	67 (53.6%)	
Female, n (%)	44 (36.1%)	50 (41.7%)	58 (46.4%)	
BMI (kg/m^2)	25.2 (22.6, 27.1)	26.3 (23.2, 30.0)	26.5 (23.3, 30.4)	0.015
SBP (mmHg)	127 (120, 140)	133 (123, 145)	127 (120, 141)	0.030
DBP (mmHg)	80 (73, 88)	81 (76, 89)	78 (72, 85)	0.079
Current smoking, n (%)	10 (8.2%)	11 (9.2%)	12 (9.6%)	0.93
Alcohol consumption (g/day)				0.004
0/rarely, n (%)	84 (68.9%)	84 (70%)	99 (79.2%)	
0.1-10, n (%)	20 (16.4%)	28 (23.3%)	19 (15.2%)	
10-30, n (%)	16 (13.1%)	7 (5.8%)	2 (1.6%)	
>=30, n (%)	2 (1.6%)	1 (0.8%)	5 (4%)	
Diabetes, n (%)	29 (23.8%)	30 (25%)	45 (36%)	0.063
History of cardiovascular disease, n (%)	6 (4.9%)	9 (7.5%)	13 (10.4%)	0.27
Blood glucose-lowering drugs, n (%)	19 (15.6%)	24 (20%)	34 (27.2%)	0.077
Lipid-lowering drugs, n (%)	18 (14.8%)	37 (30.8%)	31 (24.8%)	0.012
Antihypertensives, n (%)	39 (32%)	62 (51.7%)	66 (52.8%)	0.001
Etiology, n (%)				0.025
Viral	12 (9.8%)	15 (12.5%)	10 (8%)	
MASLD	5 (4.1%)	13 (10.8%)	16 (12.8%)	
Cholestatic liver diseases	35 (28.7%)	26 (21.7%)	39 (31.2%)	
Alcohol	10 (8.2%)	19 (15.8%)	19 (15.2%)	
Storage diseases	17 (13.9%)	7 (5.8%)	9 (7.2%)	
Autoimmune hepatitis	5 (4.1%)	5 (4.2%)	9 (7.2%)	
Others	38 (31.1%)	35 (29.2%)	23 (18.4%)	

	T1 <339 μmol/L (N=122)	T2 339-414 μmol/L (N=120)	T3 >414 μmol/L (N=125)	P value
Immunosuppressants				
Calcineurin inhibitors, n (%)	94 (77%)	86 (71.7%)	81 (64.8%)	0.104
Tacrolimus, n (%)	81 (66.4%)	75 (62.5%)	71 (56.8%)	0.30
Cyclosporine, n (%)	13 (10.7%)	11 (9.2%)	11 (8.8%)	0.87
Antimetabolite agents, n (%)	64 (52.5%)	58 (48.3%)	66 (52.8%)	0.74
Mycophenolate, n (%)	27 (22.1%)	33 (27.5%)	45 (36%)	0.052
Azathioprine, n (%)	37 (30.3%)	26 (21.7%)	21 (16.8%)	0.038
Corticosteroids, n (%)	53 (43.4%)	54 (45%)	59 (47.2%)	0.84
Prednisolone, n (%)	46 (37.7%)	44 (36.7%)	44 (35.2%)	0.92
Prednisone, n (%)	7 (5.7%)	10 (8.3%)	15 (12%)	0.22
Sirolimus, n (%)	6 (4.9%)	13 (10.8%)	27 (21.6%)	< 0.001
Total cholesterol (mmol/L)	4.0 (3.5, 4.4)	4.1 (3.7, 4.9)	4.6 (3.8, 5.2)	< 0.001
LDL cholesterol (mmol/L)	2.0 (1.6, 2.4)	2.1 (1.8, 2.5)	2.3 (1.8, 2.7)	0.002
HDL cholesterol (mmol/L)	1.4 (1.2, 1.7)	1.3 (1.1, 1.6)	1.3 (1.0, 1.7)	0.033
Triglycerides (mmol/L)	1.1 (0.8, 1.6)	1.3 (1.0, 1.8)	1.6 (1.2, 2.1)	< 0.001
Albumin (g/L)	45 (43, 47)	44.5 (43, 46)	43 (41, 45)	< 0.001
HbA1c (%)	5.1 (4.9, 5.5)	5.5 (5.2, 6.1)	5.7 (5.3, 6.3)	< 0.001
Serum creatinine (μmol/L)	82.3 (69.4, 99.0)	90.0 (76.0, 110.8)	95.9 (79.3, 114.5)	< 0.001
eGFR (mL/min/1.73 m^2)	89.2 ± 20.6	76.1 ± 25.6	69.8 ± 25.4	< 0.001
Total bilirubin (μmol/L)	13 (9, 18)	10 (7, 13)	9 (7, 12)	< 0.001
ALT (U/L)	23 (18, 33)	25 (18, 32)	28 (19, 50.8)	0.060
AST (U/L)	25.5 (20.8, 32)	25 (20, 31.8)	25.5 (21, 44.3)	0.36
GGT (U/L)	32.5 (18, 58)	36.5 (21, 72)	58 (29, 179.3)	< 0.001
ALP (U/L)	82 (60, 101)	85 (69, 102.8)	102.5 (79, 193.3)	< 0.001
Fasting glucose (mmol/L)	5.5 (5.1, 6.4)	5.7 (5.2, 6.7)	6.0 (5.5, 7.6)	0.001
Hemoglobin (mmol/L)	8.9 ± 1.1	8.8 ± 1.1	8.0 ± 1.1	< 0.001
Thrombocytes ($*10^9$/L)	168 (127, 207)	214.5 (160.5, 246)	230 (181, 281)	< 0.001
Leucocytes ($*10^9$/L)	5.3 (4.3, 6.7)	6.1 (5, 7.5)	6.9 (5.5, 8.7)	< 0.001
hs-CRP (mg/L)	1 (0.5, 1.9)	1.9 (0.8, 3.3)	5.5 (2.8, 13)	< 0.001

Data are presented as mean ± SD, median (IQR) or as proportions (n) with corresponding percentages (%). BMI: body mass index; SBP: systolic blood pressure; DBP: diastolic blood pressure; MASLD: metabolic associated steatohepatitis liver disease; LDL: low-density lipoprotein cholesterol; HDL: high-density lipoprotein cholesterol; HbA1c: hemoglobin A1c; eGFR: estimated glomerular filtration rate; ALT: alanine aminotransferase; AST: aspartate aminotransferase; GGT: gamma-glutamyl transferase; ALP: alkaline phosphatase; hs-CRP: high-sensitivity C-reactive protein.

Supplemental Table S2 Univariable and multivariable linear regression analyses showing associations between GlycA and hs-CRP with relevant clinical and laboratory parameters in patients with cirrhosis

	GlycA				hs-CRP			
	**Univariable analysis**		**Multivariable analysis**		**Univariable analysis**		**Multivariable analysis**	
	**St.β**	***P*-value**	**St.β**	***P*-value**	**St.β**	***P*-value**	**St.β**	***P*-value**
Age	-0.138 (-0.312, 0.035)	0.12			0.002 (-0.217, 0.222)	0.98		
Male	0.075 (-0.100, 0.250)	0.40			0.014 (-0.413, 0.470)	0.90		
BMI	-0.268 (-0.437, -0.099)	**0.002**			-0.135 (-0.359, 0.079)	0.21		
Smoking	-0.106 (-0.292, 0.100)	0.34			-0.091 (-0.295, 0.140)	0.48		
Lipid-lowering drugs	0.078 (-0.107, 0.253)	0.42			0.017 (-0.213, 0.248)	0.88		
Glucose-lowering drugs	-0.110 (-0.280, 0.075)	0.26			-0.119 (-0.344, 0.104)	0.29		
Antihypertensives	0.027 (-0.155, 0.205)	0.78			0.041 (-0.187, 0.270)	0.72		
Etiology								
Storage diseases	Ref				Ref			
Autoimmune hepatitis	0.040 (-0.985, 1.282)	0.80			0.171 (-0.770, 2.035)	0.37		
Cholestatic liver	0.421	0.063			0.233	0.38		

disease	(-0.053, 1.976)				(-0.697, 1.828)			
Viral	0.114 (-0.715, 1.497)	0.49			0.046 (-1.194, 1.481)	0.83		
Alcohol	0.036 (-0.937, 1.112)	0.87			0.226 (-0.734, 1.761)	0.42		
MASLD	0.232 (-0.486, 1.543)	0.30			0.304 (-0.533, 1.983)	0.26		
Vascular	0.105 (-0.751, 1.958)	0.38			0.060 (-1.263, 1.841)	0.71		
Billiary Atresia	0.154 (-0.490, 2.080)	0.22			0.059 (-1.332, 1.986)	0.70		
Child-Turcotte-Pugh classification								
A	Ref				Ref			
B	-0.208 (-0.861, 0.033)	0.069			0.081 (-0.344, 0.668)	0.53		
C	-0.087 (-0.680, 0.300)	0.44			0.454 (0.432, 1.532)	**0.001**		
MELD score	0.082 (-0.093, 0.257)	0.35			0.467 (0.277, 0.651)	**<0.001**	0.363 (0.153, 0.568)	**0.001**
Total cholesterol	0.356 (0.192, 0.520)	**<0.001**			-0.106 (-0.307, 0.102)	0.32		
HDL cholesterol	-0.431 (-0.590, -0.273)	**<0.001**			-0.523 (-0.724, -0.351)	**<0.001**		
LDL cholesterol	0.466 (0.311, 0.621)	**<0.001**	0.214 (0.050, 0.412)	**0.013**	-0.099 (-0.329, 0.120)	0.36		

Triglycerides	0.428 (0.269, 0.587)	**<0.001**	0.427 (0.270, 0.601)	**<0.001**	0.126 (-0.090, 0.355)	0.24		
ALT	0.007 (-0.205, 0.219)	0.95			0.208 (-0.004, 0.426)	0.055		
AST	-0.041 (-0.253, 0.171)	0.70			0.178 (-0.036, 0.396)	0.10		
GGT	0.267 (0.058, 0.467)	**0.012**			-0.030 (-0.249, 0.189)	0.79		
ALP	0.338 (0.133, 0.532)	**0.001**			0.145 (-0.070, 0.364)	0.18		
Fasting glucose	-0.078 (-0.290, 0.142)	0.50			0.009 (-0.205, 0.221)	0.94		
HbA1c	-0.059 (-0.335, 0.216)	0.67			-0.156 (-0.417, 0.114)	0.26		
hs-CRP	0.383 (0.181, 0.563)	**<0.001**	0.255 (0.071, 0.415)	**0.006**				
GlycA					0.383 (0.192, 0.598)	**<0.001**	0.298 (0.104, 0.510)	**0.003**

Variables with which GlycA and hs-CRP were associated in univariable analysis were included in multivariable analysis. St.β: Standardized Beta Coefficient; BMI: body mass index; MASLD: metabolic dysfunction-associated steatotic liver disease; MELD: model for end-stage liver disease; HDL: high-density lipoprotein; LDL: low-density lipoprotein; ALT: alanine aminotransferase; AST: aspartate aminotransferase; GGT: gamma-glutamyl transferase; ALP: alkaline phosphatase; HbA1c: hemoglobin A1c; hs-CRP: high-sensitivity c-reactive protein.

Supplemental Table S3 Univariable and multivariable linear regression analyses showing associations between GlycA and hs-CRP with relevant clinical and laboratory parameters in liver transplant recipients

	**GlycA**				**hs-CRP**			
	**Univariable analysis**		**Multivariable analysis**		**Univariable analysis**		**Multivariable analysis**	
	**St.β**	***P*-value**	**St.β**	***P*-value**	**St.β**	***P*-value**	**St.β**	***P*-value**
Age	0.271 (0.171, 0.370)	**<0.001**	0.098 (0.008, 0.189)	**0.032**	0.088 (-0.016, 0.193)	0.098		
Male	0.052 (-0.050, 0.155)	0.32			-0.022 (-0.258, 0.167)	0.67		
BMI	0.172 (0.070, 0.273)	**0.001**			0.164 (0.060, 0.267)	**0.002**		
Smoking	0.018 (-0.085, 0.121)	0.34			-0.046 (-0.150, 0.059)	0.39		
Lipid-lowering drugs	0.104 (0.002, 0.207)	**0.046**			-0.029 (-0.134, 0.076)	0.59		
Glucose-lowering drugs	0.150 (0.049, 0.252)	**0.004**			0.149 (0.045, 0.255)	**0.005**		
Antihypertensives	0.158 (0.057, 0.260)	**0.002**			0.101 (-0.004, 0.205)	0.059		
Etiology	-0.079 (-0.182, 0.023)	0.13			-0.079 (-0.182, 0.023)	0.13		
Storage diseases	Ref				Ref			
Autoimmune hepatitis	0.071 (-0.232, 0.872)	0.26			-0.063 (-0.835, 0.281)	0.33		

Cholestatic liver diseases	0.079 (-0.207, 0.563)	0.36			-0.162 (-0.752, 0.028)	0.069
Viral	-0.013 (-0.504, 0.414)	0.85			-0.159 (-1.000, -0.060)	**0.027**
Alcohol	0.085 (-0.180, 0.687)	0.25			-0.146 (-0.874, 0.010)	0.056
MASLD	0.224 (0.304, 1.241)	**0.001**	0.109 (0.079, 0.686)	**0.014**	-0.044 (-0.627, 0.327)	0.54
Malignancy	0.113 (0.000, 1.384)	0.050			0.115 (-0.001, 1.456)	0.050
Biliary Atresia	-0.047 (-0.813, 0.355)	0.44			-0.048 (-0.841, 0.365)	0.44
Other metabolic diseases	-0.049 (-1.234, 0.468)	0.38			-0.086 (-1.525, 0.195)	0.13
Acute and subacute liver failure	-0.042 (-0.756, 0.367)	0.50			-0.103 (-1.057, 0.100)	0.11
Polycystic liver disease	0.031 (-0.461, 0.795)	0.60			-0.115 (-1.392, 0.006)	0.052
Others	-0.044 (-0.626, 0.318)	0.52			-0.166 (-1.045, -0.091)	**0.020**
Use of tacrolimus	-0.131 (-0.233, -0.029)	**0.012**			-0.082 (-0.186, 0.023)	0.13
Use of cyclosporine	-0.060 (-0.162, 0.043)	0.26			-0.002 (-0.108, 0.104)	0.98
Use of mycophenolate	0.118 (0.016, 0.220)	**0.024**			0.090 (-0.015, 0.196)	0.093

Use of azathioprine	-0.112 (-0.214, -0.010)	**0.032**			-0.090 (-0.193, 0.015)	0.092
Use of prednisolone	-0.048 (-0.151, 0.055)	0.36			-0.095 (-0.200, 0.010)	0.075
Use of prednisone	0.116 (0.014, 0.219)	**0.026**			0.116 (0.012, 0.217)	**0.029**
Use of sirolimus	0.255 (0.156, 0.355)	**<0.001**	0.083 (0.001, 0.165)	**0.046**	0.114 (0.010, 0.219)	**0.032**
Total cholesterol	0.302 (0.204, 0.400)	**<0.001**			0.124 (0.020, 0.230)	**0.020**
HDL cholesterol	-0.127 (-0.229, -0.025)	**0.015**			0.045 (-0.060, 0.149)	0.40
LDL cholesterol	0.186 (0.084, 0.287)	**<0.001**	0.109 (0.024, 0.195)	**0.012**	-0.114 (-0.217, -0.009)	**0.033**
Triglycerides	0.231 (0.131, 0.331)	**<0.001**			-0.035 (-0.141, 0.070)	0.51
ALT	0.195 (0.092, 0.297)	**<0.001**	0.140 (0.016, 0.256)	**0.027**	0.122 (0.018, 0.225)	**0.022**
AST	0.192 (0.089, 0.293)	**<0.001**	-0.246 (-0.382, -0.096)	**0.001**	0.142 (0.038, 0.245)	**0.007**
GGT	0.346 (0.247, 0.442)	**<0.001**			0.215 (0.112, 0.316)	**<0.001**
ALP	0.479 (0.385, 0.568)	**<0.001**	0.377 (0.256, 0.482)	**<0.001**	0.326 (0.227, 0.425)	**<0.001**
Fasting glucose	0.137 (0.031, 0.243)	**0.011**	-0.137 (-0.259, -0.013)	**0.030**	0.087 (-0.020, 0.197)	0.11

HbA1c	0.235 (0.133, 0.337)	**<0.001**	0.192 (0.063, 0.321)	**0.004**	0.170 (0.066, 0.275)	**0.001**		
hs-CRP	0.574 (0.486, 0.657)	**<0.001**	0.409 (0.316, 0.480)	**<0.001**				
GlycA					0.574 (0.489, 0.662)	**<0.001**	0.526 (0.416, 0.647)	**<0.001**

Variables with which GlycA was associated in univariable analysis were included in multivariable analysis. St.β: Standardized Beta Coefficient; BMI: body mass index; MASLD: metabolic dysfunction-associated steatotic liver disease; HDL: high-density lipoprotein; LDL: low-density lipoprotein; ALT: alanine aminotransferase; AST: aspartate aminotransferase; GGT: gamma-glutamyl transferase; ALP: alkaline phosphatase; HbA1c: hemoglobin A1c; hs-CRP: high-sensitivity c-reactive protein.

Supplemental Table S4 Cox regression analyses for associations between plasma GlycA and hs-CRP levels and the risk of all-cause mortality in patients with cirrhosis and liver transplant recipients with hs-CRP below 10 mg/L.

	**GlycA, per 1 SD increment**		**hs-CRP, per 1 SD increment**	
	**HR [95%CI]**	***P* value**	**HR [95%CI]**	***P* value**
**Cirrhotic patients (n = 43; 6 deaths)**				
Model 1	1.61 [0.59-4.38]	0.348	1.48 [0-5403.09]	0.925
Model 2	1.75 [0.54-5.71]	0.353	1.96 [0-15070.6]	0.883
Model 3	1.75 [0.53-5.81]	0.357	0.90 [0-5620.14]	0.981
**LTRs (n = 305; 25 deaths)**				
Model 1	1.75 [1.06-2.90]	**0.029**	4.11 [0.36-46.23]	0.253
Model 2	1.40 [0.80-2.44]	0.237	1.50 [0.10-22.11]	0.767
Model 3	1.46 [0.77-2.74]	0.243	0.66 [0.03-14.30]	0.793

Model 1, crude model. Model 2, model 1 + with adjustment for age and sex. Model 3, model 2 with adjustment for hs-CRP (for GlycA) or GlycA (for hs-CRP). hs-CRP: high-sensitivity C-reactive protein.
